# Metataxonomics Characterization of Soil Microbiome Extraction Method Using Different Dispersant Solutions

**DOI:** 10.3390/microorganisms13040936

**Published:** 2025-04-18

**Authors:** David Madariaga-Troncoso, Isaac Vargas, Dorian Rojas-Villalta, Michel Abanto, Kattia Núñez-Montero

**Affiliations:** 1 Facultad de Ciencias de La Salud, Instituto de Ciencias Aplicadas, Universidad Autónoma de Chile, Temuco 4810101, Chile; david.madariaga@cloud.uautonoma.cl; 2 Escuela de Biología, Instituto Tecnológico de Costa Rica, Cartago 30101, Costa Rica; isaacvv131200@gmail.com; 3 Cellular and Molecular Biology Research Center, Universidad de Costa Rica, San José 11501, Costa Rica; rojasvillaltadorian@gmail.com; 4 Núcleo Científico y Tecnológico en Biorecursos (BIOREN), Universidad de La Frontera, Avenida Francisco Salazar, Temuco 4811230, Chile

**Keywords:** microbiome transplantation, soil bioremediation, metataxonomics, dispersant solutions, 16S rRNA gene metataxonomics, metabarcoding

## Abstract

Soil health is essential for maintaining ecosystem balance, food security, and human well-being. Anthropogenic activities, such as climate change and excessive agrochemical use, have led to the degradation of soil ecosystems worldwide. Microbiome transplantation has emerged as a promising approach for restoring perturbed soils; however, direct soil transfer presents practical limitations for large-scale applications. An alternative strategy involves extracting microbial communities through soil washing processes, but its success highly depends on proper microbiota characterization and efficient extraction methods. This study evaluated a soil wash method using four different dispersant solutions (Tween-80, NaCl, sodium citrate, and sodium pyrophosphate) for their ability to extract the majority of microbial cells from Antarctic and Crop soils. The extracted microbiomes were analyzed using 16S rRNA gene metataxonomics to assess their diversity and abundance. We found that some treatments extracted a greater proportion of specific taxa, and, on the other hand, some extracted a lower proportion than the control treatment. In addition, these dispersant solutions showed the extraction of the relevant microbial community profile in soil samples, composed of multiple taxa, including beneficial bacteria for soil health. Our study aims to optimize DNA extraction methods for microbiome analyses and to explore the use of this technique in various biotechnological applications. The results provide insights into the effect of dispersant solutions on microbiome extractions. In this regard, sodium chloride could be optimal for Antarctic soils, while sodium citrate is suggested for the Crop soils.

## 1. Introduction

Microbes are fundamental to soil functioning and health, driving critical processes such as nutrient cycling, organic matter decomposition, and plant growth promotion [1,2,3]. The accurate characterization of microbial communities is essential for advancing our understanding of these processes and developing strategies to maintain or restore soil health. Among the various strategies for managing microbial communities and studying soil properties, soil microbiome transplantation has emerged as an innovative technique. This approach involves transferring soil between regions, enabling the investigation of physical, chemical, and biological changes driven by soil microbiota under natural conditions [4,5]. Its application has demonstrated significant potential for restoring degraded ecosystems and mitigating climate change effects by enhancing microbial biodiversity and improving soil health [6,7]. Notably, studies have shown that soil microbial communities can adapt to new local conditions, effectively overcoming historical constraints within relatively short timeframes [6]. Furthermore, global analyses reveal that soil transplantation accelerates vegetation recovery by up to 40% compared to alternative methods, underscoring its efficacy [8]. These findings highlight the promise of soil microbiome transplantation as a critical tool in international ecological restoration initiatives [9,10].

The direct transfer of soil has been evaluated in controlled experiments, but this method presents clear practical limitations for large-scale applications [11,12]. An alternative strategy involves extracting microbial communities through a soil washing process. However, extracting soil microbial cells is challenging due to the complex and heterogeneous nature of these matrices, hindering the efficiency and representativeness of microbiome analyses [13,14,15]. One key step in the extraction of soil microbiomes is the use of dispersant solutions (e.g., surfactants, chemical agents) that facilitate the separation of microbial cells from soil particles [16]. Different dispersants can vary in their effectiveness at releasing microbes into suspension, influencing the diversity and abundance of microorganisms that are ultimately analyzed [17]. This variability poses a concern for soil microbiome studies. It may introduce biases that affect the interpretation of microbial community structure and function.

Dispersant solutions have a critical role in microbiome extraction due to their high potential regarding soil wash microbiome transplants approaches. However, there is a lack of comprehensive studies evaluating their impact on extraction efficiency and microbial diversity, particularly using high-resolution techniques like metataxonomics. Understanding these effects is crucial for standardizing extraction protocols and ensuring that soil microbiome analyses are representative of the *in situ* microbial communities.

In this study, we assessed the effect of four different dispersant solutions—Tween-80, sodium chloride, sodium citrate, and sodium pyrophosphate—on the extraction of microbial communities from Antarctic and Crop soils. We employed metataxonomics to evaluate differences in microbial diversity and abundance associated with each dispersant treatment. This aimed to identify which dispersant enables a more representative extraction of soil microbiomes and to assess whether a soil wash effectively retains the representative microbial community of the donor sample. We hope to provide evidence on methods for soil microbiome research, particularly for extraction protocols in microbiome transplantation, which might lead to future research on soil restoration in the face of global environmental challenges.

## 2. Materials and Methods

### 2.1. Collection and Processing of Antarctic and Crop Soil Samples

Antarctic soil samples were collected on King George Island, South Shetland Islands (62°9′44″ S, 58°28′9″ W), during a scientific expedition in January 2023. For the Crop samples, the soil was collected from a private agricultural property in the Araucanía Region, Chile (March 2023; 38°44′03.1″ S, 72°15′41.7″ W). Both samples were collected from each sampling site at a depth of 5–10 cm from 5 collection points randomly selected from a surface of 150 m^2^. A total of two kilograms of soil were stored in sterile bags, kept in a cooler, and directly taken to the laboratory. The samples were later homogenized, and subsamples from each soil were transferred to 50 mL sterile tubes for storage at 4 °C until further use. Three subsamples per sample site were used after 15–20 days from collection, when those were subjected to microbial extraction, DNA extraction, and soil chemical characterization.

### 2.2. Soil Chemical Chracterization

Chemical analyses were conducted at the SmartC platform of the BIOREN Research Center, Universidad de La Frontera (Temuco, Chile) using 500 g of the soil sample. The following chemical parameters were determined according to the official guidelines of the Chilean Society of Soil Science (CNA standards); pH (in water and in CaCl_2_), organic matter (% by oxidation of dichromates), the availability of phosphorus P (Olsen method), exchange cations on ammonium acetate (Ca, Mg, K, Na), and exchangeable Al were extracted with 1 mol/L KCl. The 1 mol/L ammonium acetate (CH_3_COONH_4_) solution was adjusted to pH 4.8 for quantizing extractable Al, cation exchange capacity (CICE), saturation of bases (S.Bases), and micronutrients B, Zn, Cu, Fe, Mn, and S (extracted using DTPA). The sum of exchangeable bases plus Al was used to calculate the cation exchange capacity (CICE).

### 2.3. Use of Dispersant Solutions for Microbial Extractions

To evaluate the effect of dispersants on microbiome extraction, four different solutions were prepared: Tween-80 (0.1%), NaCl (0.8%), sodium citrate (0.5%), and sodium pyrophosphate (0.2%). These dispersants and their specific concentrations were chosen based on established protocols in the literature that demonstrate both their safety for microbial cells and proven effectiveness in separating microbial communities from soil particles, therefore facilitating the extraction of a representative microbiome [17,18,19]. For each dispersant, 6 g of soil were added to 24 mL of the respective solution. The assays which followed were performed in triplicates.

Microbial extraction from soil particles was based on the method described in Durán et al., 2022 [19], with some modifications (Figure S1). Briefly, the samples were horizontally shaken in vortex for 15 min at medium speed. Then they were centrifuged at 280 g for one minute. The supernatant was transferred to a new sterile tube, and a second centrifugation was carried out at 3000 g for 30 min to obtain a pellet with the microbial cells. The pellet was recovered, washed, and resuspended using 1 mL of saline solution (0.8% NaCl) into cryovials (transplant), and the supernatant was discarded.

Subsequently, one µL of the transplant was diluted in 999 µL of saline solution (and culture by extension) in Petri dishes with nutrient agar for the counting of total aerobes. The plates were incubated at room temperature (25 °C) for 36 h for Crop soils and for 7 days for Antarctic soil samples. After incubation, colony forming units (CFU) were manually counted.

### 2.4. DNA Extraction

DNA extraction was performed using the DNeasy PowerSoil Pro Kit (Qiagen, Hilden, Germany) according to the manufacturer’s protocol with the following modifications: (i) 250 mg of respective donor soil was employed as the control of microbial profiling (Control); (ii) For transplant treatments’ DNA extraction, the input sample was 33.3 µL of the transplant; (iii) All the samples were shaken for 10 min in the lysis step; and (iv) DNA was eluted in 50 µL of C6 solution. DNA quality and quantity were assessed using a Nanoquant spectrophotometer and Quantus fluorometer (Promega, Madison, WI, USA), with all samples meeting the standard quality threshold (A260/A280 ratio of 1.8–2.0). DNA integrity was verified by gel electrophoresis prior to downstream applications.

### 2.5. Metataxonomics Library Preparation and Sequencing

16S rRNA gene amplification and sequencing was performed with the 16S Barcoding SQK-RAB204 Kit (Oxford Nanopore Technologies, Oxford, UK), following the manufacturer’s instructions. Briefly, the extracted DNA was adjusted to 8.5 ng in 10 µL and combined with 10 µL of nuclease-free water. A master mix was prepared using 25 µL of LongAmp Hot Start Taq 2X (Oxford Nanopore Technologies, Oxford, UK), and 10 µL of the barcodes were used for each of the samples. Nuclease free water was used as a negative control. The PCR was carried out in the MiniAmpPlus Thermal Cycler (Applied Biosystems, Waltham, MA, USA) with the following cycle conditions: (i) initial denaturation at 95 °C for 1 min and 1 cycle, (ii) the second denaturation at 95 °C for 20 s for 35 cycles, (iii) the annealing at 55 °C for 30 s for 35 cycles, (iv) the extension at 65 °C for 2 min for 35 cycles, and (v) the final extension at 65 °C for 5 min and 1 cycle. Amplified DNA was purified using a magnetic rack and AMPure XP beads as recommended by the manufacturer. All barcoded libraries were then pooled in an equal ratio to complete 100 fmoles of DNA in 10 µL of stabilizing buffer (10 mM Tris-HCl, pH 8.0, 50 mM NaCl), and 1 µL of RAP solution was added. Complete 16S rRNA gene sequencing was performed using the MinION Mk1C platform with R9.4.1 flow cells and MinKNOW v26.06 (Nanopore Oxford Technologies, Oxford, UK). All sequences used in this study are available at the National Center for Biotechnology Information (NCBI), under the BioProject accession: PRJNA1185422.

### 2.6. Microbiota Profiling and Statistical Analysis

Raw reads were filtered and trimmed using chopper v0.7.0 with a quality score threshold of 9, minimum length of 1000 bp, and maximum length of 1900 bp. Adapters were removed using Porechop v0.2.4 with a middle adapter threshold of 85% and discarding reads with middle adapters. Taxonomic classification was performed with Centrifuge v1.0.4 [20] using quality-aware alignment mode and a minimum read length of 800 bp against the SILVA v138 database [21]. Then, the MicrobiomeAnalyst 2.0 [22] webserver (https://www.microbiomeanalyst.ca/) was employed to evaluate diversity indexes and statistical analyses. Crop and Antarctic soil samples were rarefied to a library size of 4658 reads and 4816 reads, respectively. Dataset normalization was conducted using the Total Sum Scaling (TSS) method, available at the same webserver.

Alpha diversity indexes were calculated in marker data profiling (MDP) to generate Chao1 and Shannon indexes at the genus level. Statistical differences between groups were evaluated using the Mann–Whitney/Kruskal–Wallis test with post-hoc pairwise comparisons. Beta diversity was assessed through Principal Coordinates Analysis (PCoA) based on the Bray–Curtis dissimilarity matrix at each taxonomic level. Statistical significance was determined using PERMANOVA and PERMDISP with pairwise comparisons with Benjamini–Hochberg correction. Redundancy analysis (RDA) was performed using the vegan package in R to assess the relationship between microbial community composition and explanatory variables (soil type and dispersant treatment). Data were Hellinger-transformed prior to analysis. Distance-based RDA (dbRDA) was also conducted using Bray–Curtis dissimilarities. Statistical significance was determined by permutation tests (999 permutations). To identify microbial communities with significant changes (*p* < 0.05) across treatments, Linear Discriminant Analysis Effect Size (LEfSe) was performed among different taxonomic levels. The Log LDA score threshold was set to 0.2 with a False Discovery Rate (FDR) adjusted based on the *p*-values of each taxa. All programs were run under defaults parameters, except when otherwise specified.

## 3. Results and Discussion

**Figure 1:**
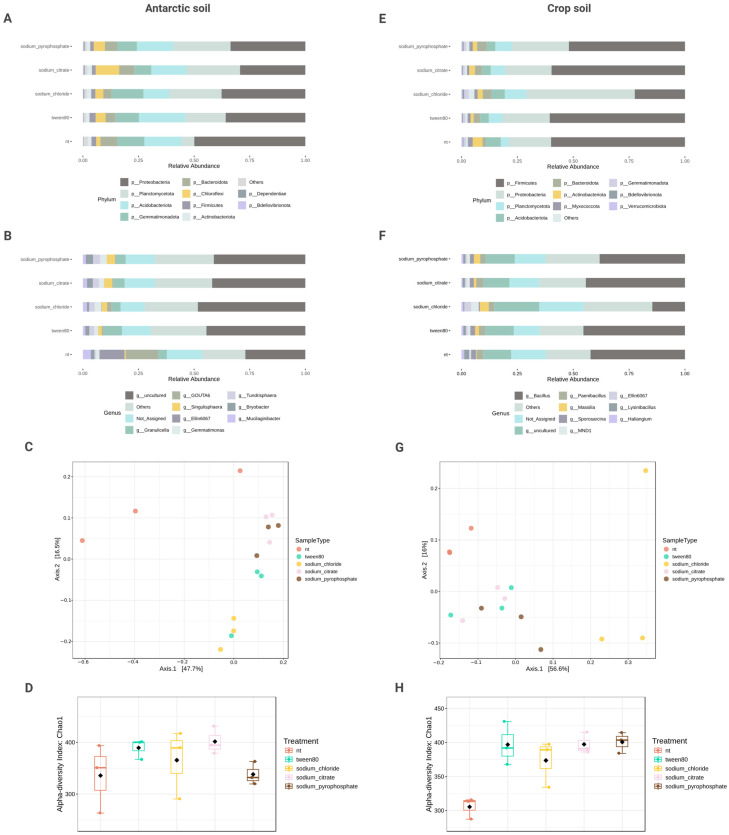
Taxonomic composition and diversity analyses of Antarctic and Crop soil microbiomes extracted using different dispersant solutions. (**A**,**E**) Relative abundance of bacterial phyla in Antarctic and Crop soils, respectively. (**B**,**F**) Relative abundance at genus level in Antarctic and Crop soils, respectively. (**C**,**G**) Principal Coordinates Analysis (PCoA) based on Bray–Curtis dissimilarity matrix showing beta diversity of microbial communities in Antarctic and Crop soils, respectively. (**D**,**H**) Alpha diversity analysis using Chao1 index for Antarctic and Crop soils, respectively. Treatments included no treatment control (nt), Tween-80, sodium chloride, sodium citrate, and sodium pyrophosphate. Data show differences in microbial composition and diversity across treatments.

The bacterial profile characterization showed the presence of several phyla previously reported in both types of soil environments [23,24,25,26]. Antarctic soil had a major abundance of the Proteobacteria (61.2%), Planctomycetota (33.31%), and Acidobacteriota (25.65%) phylum; whilst Crop soil was dominated by Bacillota (67.49%, formerly Firmicutes), followed by Proteobacteria (48.77%) and Planctomycetota (11.35%) (Figure 1A,E). Bacillota is a common phylum in soils; although its presence in the Antarctic has been stated [27], this increased percentage in our Crop samples might be related to low organic matter and high inorganic fertilization [28]. In addition, the reduced abundance of Bacillota in the Antarctic sampling site (Deception Island) has been previously reported [29].

The main genera found in the Antarctic samples were composed of *Granulicella* (11.23%), GOUTA6 (a group from the Nitrosomonadaceae familiy) (24.73%), *Gemmatimonas* (3.76%), *Singulisphaera* (4.59%), Ellin6067 (Candidatus *Solibacter usitatus*, 18.35%), *Bryobacter* (3.48%)*,* and *Mucilaginibacter* (3.36%). These genera have been previously reported for this environment [30,31,32,33,34,35,36,37] (Figure 1B). Particularly, several species of *Granulicella* are associated with the metabolism of diverse polysaccharides, resilience to fluctuating extreme temperatures, and tolerance to aluminum stress [38,39]. *Gemmatimonas* has demonstrated molecular nitrogen reduction properties favoring nitrogen accessibility to plants in nutrient-deficient soils [40]. *Singulisphaera* is considered part of the core taxa of healthy soils able to suppress diseases and stabilize microbial networks [41]. Finally, *Mucilaginibacter* species are thought to be plant-growth-promoting bacteria [42]. These results indicate that the Antarctic samples are composed of beneficial bacteria to the soil environment in both the control (nt) and dispersant-treated samples. As mentioned above, the optimization of extraction protocols for microbiome extraction is a relevant research area for soil transplantation treatments. To our knowledge, no previous studies have evaluated the effect of dispersant on microbiome extractions. Thus, our research pioneers in this regard, stating the extraction of beneficial bacteria for soil transplantation assays.

Moreover, the profiling of Crop soil showed the dominance of the genera *Bacillus* (49.19%), *Paenibacillus* (3.14%), *Massilia* (5.48%), *Sporosarcina* (2.27%), *Lysinibacillus* (2.19%), *Haliangium* (1.29%), Ellin6067 (4.87%), and MND1 (a group from the Proteobacteria phylum, 3.38%) (Figure 1F). These genera are known to be part of the core microbiota of soil samples (e.g., *Bacillus*, *Haliangium*) [36,43,44,45,46,47,48]. In this sense, most have been used for bioremediation of soil contaminated with heavy metals, herbicides, and pesticides (e.g., *Paenibacillus*, *Massilia*, *Sporosarcina*, *Lysinibacillus*) [49,50,51,52]. This result was expected as the soil sample comes from an agricultural site with recurrent agrochemicals usage. Hence, we consider that the microbial communities from this environment have shifted towards taxa able to tolerate or degrade these compounds.

In addition, a significant proportion of the features in the Antarctic samples were not identified (>25%). The Antarctic environment with its poly-extreme conditions led to fast speciation of microbes due to the increased rate of horizontal gene transfer in order to adapt to the hazardous conditions [53,54]. Therefore, some studies have proposed that the Antarctic environment comprises a large percentage of unknown and still-uncultured species, up to 50% [55], a hypothesis supported by the results presented here.

Our results showed that the use of dispersant solutions for microbiome extractions has a subtle effect on taxonomic composition, at all taxonomic levels studied, for both Antarctic and Crop soils. Also, the differential physical and chemical properties of Antarctic and Crop soils could likely affect the amount and variety of microorganisms we can recover through the preparation of a microbial extract. Antarctic soil is sandy loam and showed significant cation exchange capacity and semi-arid soil quality with good minerals but low organic matter (2%) and no or little available nutrients (e.g., P and K) (Table S1) which may limit microbial biomass. Even so, its near-neutral pH (7.17 in water, 5.99 in CaCl_2_) is likely to favor a wide range of microbes, including oligotrophic and stress-tolerant strains that are usually of high biotechnological interest due to their hardiness and unique metabolic abilities. Unlike Antarctic soil, Crop soil is silty clay loam and had a higher abundance of macronutrient potassium and micronutrients Zn, B, and Mn, which can contribute towards the richness of all kinds of microbes, particularly copiotrophs. For this reason, the soil extract yielded a greater number of microbes, which are likely involved in plant and soil interactions or nutrient cycling.

For Crop soils, almost all samples exhibited a similar microbial composition to the untreated soil at the phylum level, with the exception of sodium chloride. A similar pattern was observed at the genus level, where sodium chloride treatment led to an increased relative abundance of the Proteobacteria phylum (>40%) and uncultured or unassigned genera (>25%). These findings highlight the significant influence of soil type on the effectiveness of different dispersants in extracting a representative microbiome from the donor soil. For instance, Antarctic soil appears to harbor a microbiome subtly prone to variation during extraction, likely due to the poly-extreme environmental conditions that are considered inhospitable for many microbial species. According to Varliero et al. (2024) and Lebre et al. (2023) [25,56], Antarctic soils experience frequent freeze–thaw cycles during the austral summer, low water availability, and high UV radiation, all of which impose strong selective pressures on microbial communities. Metagenomic analysis revealed that Antarctic soil microbiomes are enriched in stress-response genes like catalase and trehalose synthase, indicating adaptation to oxidative stress and thermal shock, which may render these communities particularly sensitive to laboratory manipulations [56]. This research also demonstrated clear biogeographical regionalization of microbial communities with high spatial heterogeneity [56], which might explain why dispersant treatments had more pronounced effects on Antarctic soil samples. Such harsh conditions result in microbial communities with specialized physiological states and membrane structures that are particularly sensitive to physical and chemical manipulations during extraction, as also observed in previous studies. Such conditions exert strong selective pressures on the Antarctic microbiome, resulting in unstable communities that are particularly sensitive to disturbances [57,58,59].

Moreover, the colony-forming units (CFUs) count showed no statistical difference among most samples compared to the control group (*p* > 0.05). Only the sodium citrate treatment had significantly higher CFUs in comparison to the other Antarctic treatments (Figure S4). Hence, the use of dispersant solutions had no negative effect on the viability of the microbiome extraction. Interestingly, in Antarctic soils, sodium citrate demonstrated a higher number of colonies in comparison to sodium chloride. As observed on microbial composition, sodium chloride increases the relative abundance of unculturable taxa while sodium citrate reduces it (Figures S2–S5). Hence, the higher cultivability did not influence microbial diversity or richness significantly (alpha and beta diversity are discussed later).

In order to evaluate microbial communities with significant changes (*p* < 0.05) across treatments, we performed a Linear Discriminant Analysis (LDA) Effect Size (LEfSe) among different taxonomic levels (Tables S3 and S4). At phylum level, Abditibacteriota (*p* = 0.043, LDA = 3.15), Bacteroidota (*p* = 0.049, LDA = 4.31), and Nitrospirota (*p* = 0.049, LDA = 3.07) presented significant differences in the Antarctic samples, while Bdellovibrionota (*p* = 0.031, LDA = 3.33) showed variation in the Crop soils. For the Antarctic soil, the class level had nine taxa with significant results, including Kapabacteria (*p* = 0.017, LDA = 3.04), Phycisphaerae (*p* = 0.020, LDA = 3.37), Clostridia (*p* = 0.022, LDA = 3.85), and Nitrospiria (*p* = 0.030, LDA = 3.08). Specifically, Nitrospiria was completely absent from sodium chloride treatment. In the Crop samples, Kapabacteria (*p* = 0.016, LDA = 2.46), Alphaproteobacteria (*p* = 0.029, LDA = 4.43), and Omnitrophia (*p* = 0.038, LDA = 2.64) changed significantly among treatments. Particularly, the Gammaproteobacteria class expressed variations in both soil types. The LDA scores demonstrated that this alteration in Gammaproteobacteria had the highest scores (*p* = 0.021, LDA = 5.17, and *p* = 0.019, LDA = 5.21 for the Crop and Antarctic sample, respectively). This indicates a substantial effect of the dispersant solutions on these bacterial populations.

The order level exhibited the most differences with 21 and 15 taxa for the Antarctic and Crop samples, respectively. In the Antarctic soils, Kapabacteriales (*p* = 0.017, LDA = 3.04), Tepidisphaerales (*p* = 0.018, LDA = 3.37), Clostridiales (*p* = 0.018, LDA = 3.92), Burkholderiales (*p* = 0.019, LDA = 5.17), and Xanthomonadales (*p* = 0.019, LDA = 4.36) had significant values of *p* < 0.02. Remarkably, Staphylococcales was present in the control sample but absent in all the treatments, marking an example of how the use of dispersant might drastically modify the extracted microbiome. In particular, Staphylococcales present many heavy-metal and alkali-tolerant species that have been proposed for potential use in soil bioremediation [60,61]. Hence, the loss of this order in the dispersant treatments could thoroughly affect the posterior use in microbiome transplantations assays. Moreover, for the Crop soils, Pseudomonadales (*p* = 0.013, LAD = 4.09) and Kapabacteriales (*p* = 0.016, LAD = 2.46) showed the lowest *p*-values (*p* < 0.02) across the orders with significant differences. For both soil types, LDA scores were higher for the Burkholderiales order (LDA = 5.17 and 5.03 for the Antarctic and Crop, respectively). Burkholderiales are considered a relevant group of hydrocarbons, chemical, and heavy metal bioremediation bacterial species; thus, results are relevant for evaluating the biotechnological potential of the extracted microbiome [62,63,64]. At the genus level, several variations were observed among Crop samples compared to the Antarctic soil which presented no significantly different genus. *Frankiales* was absent in Tween-80 and sodium chloride but highly abundant in the control groups and the other dispersant-treated samples. Moreover, *Lautropia* (*p* = 0.008, LDA = 2.16) and *Sporolactobacillus* (*p* = 0.009, LDA = 2.46) had very significant differences in the Crop samples (*p* < 0.01).

Overall, these results imply that the use of dispersants has a subtle effect on the extracted microbiome from soil samples, differences that might lead to the loss of essential bacteria for enhancing soil health. In the Antarctic samples, sodium chloride treatments presented the highest abundance in taxa with significant differences, and often including bacterial groups lost with other dispersants. We report the modifications in the microbiome due to the use of particular dispersants in two soil types, highlighting an effect on the abundance of certain taxa. However, the relatively high False Discovery Rate (FDR) values (0.26–0.35) across all taxonomic levels indicate a need for cautious interpretation of these findings. These high FDR values might indicate the possibility of false positive results in the significant differences of the evaluated taxa. Commonly, FDR values above 0.2 are insufficient to conclude a significant difference in the taxonomic abundance. However, this provides a preliminary screening of the microbial variability that soil extraction might induce. To ensure the reliability of our finding, a pairwise analysis was conducted using the Benjamini–Hochberg correction that allows for the reduction of the FDR values. These results are presented with the beta diversity analysis.

To evaluate the alpha diversity of our samples, the Chao1 and Shannon indexes were calculated (Figure 1D,H; Figure S3 and Table S1). None of the treatments had a significant difference to the control group in the Chao1 (richness, *p* = 0.11 and *p* = 0.35 for Crop and Antarctic soil, respectively) or Shannon analysis (*p* = 0.07 and *p* = 0.46 for Crop and Antarctic soil, respectively). Despite observable taxonomic shifts at various levels (e.g., phylum and genus) across treatments, alpha diversity metrics such as the Chao1 and Shannon indices did not exhibit significant differences. This suggests that the overall richness and evenness of microbial communities remained stable, possibly due to a high degree of functional redundancy within the soil microbiota. Functionally redundant communities can maintain ecological roles even when taxonomic composition shifts, particularly in environments with strong selective pressures, such as Antarctic soils. Furthermore, the dominance of certain abundant taxa (e.g., *Bacillus* in Crop soil and Proteobacteria in Antarctic soil) may mask diversity changes by stabilizing richness metrics despite fluctuations in the presence of less abundant groups. These core taxa might exert a buffering effect on diversity indices. Therefore, the lack of significant change in alpha diversity reflects not an absence of microbial response, but rather a compositional turnover within a framework of functional and structural stability. These results indicate that, although some taxa might be affected using dispersants, general diversity and possibly function are not affected, and a representative similarity to the control group is extracted.

Beta diversity analysis showed a significant difference in the Bray–Curtis distances between the treated and control samples of both Crop (F-value = 4.42, R^2^ = 0.64, *p* = 0.001) and Antarctic soil (F-value = 3.28, R^2^ = 0.58, *p* = 0.001) based on the PERMANOVA (Figure 1C,G). However, the results for the variance of the samples showed no significant difference as of the PERMDISP analysis (F-value = 1.29, *p* = 0.34 and F-value = 0.48, *p* = 0.75 for Crop and Antarctic soil, respectively). The lack of significance in the pairwise analysis, but visible with the overall PERMANOVA might be related to a sample bias. All the treatments were performed using a single soil sample and the difference in taxonomic abundances is subtle and related to poorly represented species. Therefore, these factors might have induced this significance value in the general analysis, while remaining unperceived by the pairwise results. It is important to remark that to make sure these results were reliable, both the PERMANOVA and PERMDISP were conducted using the Benjamini–Hochberg correction to reduce FDR values.

Visually, it is noticeable that the treatment samples are clustered independently from the control groups, indicating a subtle difference. Meanwhile, for Crop soil, the clustering tendency is reversed. Treated and control samples are clustered nearby, excluding the sodium chloride dispersant. As mentioned above, sodium chloride seems to greatly affect the extracted microbiome of Crop soil, leading to an altered diversity. To further examine the relationship between dispersant treatments and microbial community structure, redundancy analysis (RDA) was performed (Figure S6). The RDA revealed that soil type was the primary driver of community variation (F = 87.25, *p* = 0.001), explaining 54.46% of the total variation along the first axis. Importantly, both the dispersant treatment effect (F = 3.89, *p* = 0.004) and the soil-type interaction (F = 3.84, *p* = 0.001) were statistically significant, confirming that the impact of dispersants differs between Antarctic and Crop soils. Distance-based RDA (dbRDA) using Bray–Curtis dissimilarities confirmed these findings, with significant effects of soil type (F = 47.49, *p* = 0.001), treatment (F = 2.79, *p* = 0.008), and their interaction (F = 2.63, *p* = 0.010). Based on all the discussed results, we consider sodium chloride was the optimal dispersant for microbiome extraction from the Antarctic soils. For the Crop soils, sodium citrate is suggested as it maintains bacterial composition and has high viability for culture.

While our results highlight the ability of dispersant-assisted methods to extract taxonomically representative microbial communities, we acknowledge that future studies should assess the integrity and viability of the recovered cells, functional profile, and metabolic potential. Techniques such as fluorescence-based live/dead staining and qPCR quantification of microbial biomass would help determine whether dispersants affect cell integrity or cause DNA degradation, while those such as enzymatic activity tests, metatranscriptomics, or RT-qPCR target key metabolic pathways. These analyses are critical next steps to validate the ecological and functional utility of extracted microbiomes in practical applications for microbial transplantation.

## 4. Conclusions

The use of dispersant solutions during microbiome extraction has a subtle effect on the microbial composition of the soil sample. Particularly, the unstable microbiome of the Antarctic soils generates great variation in the microbial communities. However, the sodium chloride solution presented the highest similarity to the control group. In Crop soils, the use of dispersants had no negative effect on the extracted microbiome (except for sodium chloride). However, sodium citrate had the highest cultivability, proposing an increased viability of the microbiome. Moreover, several of the solutions used provoked the loss of a significant abundance of beneficial taxa for soil bioremediation. For instance, Staphylococcales and Burkolderiales were thoroughly disturbed by the use of dispersant, leading to their complete loss or significant reduction in the treated samples, respectively. Despite the specific effect of dispersant in certain taxa groups, the overall richness and diversity had no significant differences among treatments, indicating a representative extraction of the microbiome. Overall, these results recommend the use of sodium chloride and sodium citrate for Antarctic and Crop soil extractions, respectively. In this regard, our study is a pioneer in the field of the optimization and standardization of microbiome extraction protocols, and highlights the need to confirm the microbial composition before transplantation to ensure the achievement of representative beneficial microbes. This paves the way towards the adoption of microbiome extraction protocols in several biotechnological applications, such as soil transplantations.

## Data Availability

The datasets generated and/or analyzed during the current study are available in the National Center for Biotechnology Information repository, under the BioProject PRJNA1185422 accession.

## Supplementary Materials

The following supporting information can be downloaded at: https://www.mdpi.com/article/10.3390/microorganisms13040936/s1, Figure S1. Antarctic and Agricultural soil microbiome DNA extraction protocol using different dispersant solutions. The workflow shows both control (1) and dispersant treatments (2) with four different dispersant solutions in triplicated assays, followed by sequential centrifugation steps (2.1–2.3), bacterial pellet storage (2.4), and final DNA extraction (2.5). The extracted DNA was subsequently processed for 16S rRNA metataxonomic analysis (3) using the 16S Barcoding SQK-RAB204 Kit to characterize the bacterial communities. Created using BioRender.com. Figure S2. Taxonomic composition of bacterial communities at Class, Order, and Family levels in Antarctic and Crop soils under different dispersant treatments. Relative abundance of bacterial taxa at Class, Order, and Family taxonomic ranks in Antarctic soil (left panels) and Crop soil (right panels) after extraction using different dispersant solutions: sodium pyrophosphate, sodium citrate, sodium chloride, Tween80, and no-treatment control (nt). The data illustrate the distinct taxonomic compositions between soil types and the effects of different dispersant treatments on the extracted microbial communities. This analysis complements the phylum and genus-level data shown in Figure 1. Figure S3. Alpha diversity analysis using Shannon index for Antarctic and Crop soils treated with different dispersant solutions. Shannon diversity index values for bacterial communities in Antarctic soil (left) and Crop soil (right) after treatment with different dispersant solutions (Tween-80, sodium chloride, sodium citrate, and sodium pyrophosphate) compared to no-treatment control (nt). Table S1. Chemical parameters of the soil samples included in this study. Table S2. Alpha diversity values for Crop and Antarctic soil samples. Table S3. Linear Discriminant Analysis Effect Size (LEfSe) of Antarctic soil samples. Table S4. Linear Discriminant Analysis Effect Size (LEfSe) of Crop soil samples. Figure S4. Colony-forming unit (CFU) counts in Antarctic soil samples. UFC = colony-forming units (Spanish acronym), NaCl = sodium chloride treatment, TSPP = Pyrophosphate treatment, SCTE = sodium citrate treatment. Those treatments with a significant difference (*p* < 0.05) are indicated with an asterisk (*). Figure S5: Colony-forming unit (CFU) counts in Crop soil samples. UFC = colony-forming units (Spanish acronym), NaCl = sodium chloride treatment, TSPP = Pyrophosphate treatment, SCTE = sodium citrate treatment. No significant differences were present among the treatment (*p* > 0.05). Figure S6. Redundancy Analysis (RDA) and Distance-based Redundancy Analysis (dbRDA) of microbial communities in Antarctic and Crop soils under different extraction treatments. Redundancy analysis revealed strong separation of microbial communities based on soil type (F = 87.25, *p* = 0.001), with Antarctic and Crop soil samples forming distinct clusters along the primary axis, which explained 65.1% of the variation. Treatment methods significantly influenced community structure (F = 3.89, *p* = 0.007), and we observed a significant soil type × treatment interaction (F = 3.84, *p* = 0.007). Distance-based redundancy analysis confirmed these patterns, with soil type as the dominant factor (F = 47.49, *p* = 0.001) and significant effects of treatment (F = 2.79, *p* = 0.006) and their interaction (F = 2.63, *p* = 0.007). Both ordination methods consistently demonstrated that extraction treatments affected microbial communities differently depending on soil type.

## References

[1] Delgado-Baquerizo M., Giaramida L., Reich P.B., Khachane A.N., Hamonts K., Edwards C., Lawton L.A., Singh B.K. (2016). Lack of Functional Redundancy in the Relationship between Microbial Diversity and Ecosystem Functioning. J. Ecol..

[2] Fierer N. (2017). Embracing the Unknown: Disentangling the Complexities of the Soil Microbiome. Nat. Rev. Microbiol..

[3] Jansson J.K., Hofmockel K.S. (2019). Soil Microbiomes and Climate Change. Nat. Rev. Microbiol..

[4] Heděnec P., Jílková V., Lin Q., Cajthaml T., Filipová A., Baldrian P., Větrovský T., Krištůfek V., Chroňáková A., Setälä H. (2019). Microbial Communities in Local and Transplanted Soils along a Latitudinal Gradient. Catena.

[5] Hopple A.M., Pennington S.C., Megonigal J.P., Bailey V., Bond-Lamberty B. (2022). Disturbance Legacies Regulate Coastal Forest Soil Stability to Changing Salinity and Inundation: A Soil Transplant Experiment. Soil Biol. Biochem..

[6] Sun B., Wang F., Jiang Y., Li Y., Dong Z., Li Z., Zhang X.X. (2014). A Long-Term Field Experiment of Soil Transplantation Demonstrating the Role of Contemporary Geographic Separation in Shaping Soil Microbial Community Structure. Ecol. Evol..

[7] Benetková P., van Diggelen R., Háněl L., Vicentini F., Moradi R., Weijters M., Bobbink R., Harris J.A., Frouz J. (2022). Soil Fauna Development during Heathland Restoration from Arable Land: Role of Soil Modification and Material Transplant. Ecol. Eng..

[8] Gerrits G.M., Waenink R., Aradottir A.L., Buisson E., Dutoit T., Ferreira M.C., Fontaine J.B., Jaunatre R., Kardol P., Loeb R. (2023). Synthesis on the Effectiveness of Soil Translocation for Plant Community Restoration. J. Appl. Ecol..

[9] Liang Y., Jiang Y., Wang F., Wen C., Deng Y., Xue K., Qin Y., Yang Y., Wu L., Zhou J. (2015). Long-Term Soil Transplant Simulating Climate Change with Latitude Significantly Alters Microbial Temporal Turnover. ISME J..

[10] Chang L., Sun X., Wang B., Gao M., Chen L., Liang A., Wu D. (2021). Green More than Brown Food Resources Drive the Effect of Simulated Climate Change on Collembola: A Soil Transplantation Experiment in Northeast China. Geoderma.

[11] Gowda M.T., Prasanna R., Rao U., Somvanshi V.S., Singh P.K., Singh A.K., Chawla G. (2023). Microbiome Transplant Can Effectively Manage Root-Knot Nematode Infectivity in Tomato. Appl. Soil Ecol..

[12] Yergeau E., Bell T.H., Champagne J., Maynard C., Tardif S., Tremblay J., Greer C.W. (2015). Transplanting Soil Microbiomes Leads to Lasting Effects on Willow Growth, but Not on the Rhizosphere Microbiome. Front. Microbiol..

[13] McPherson M.R., Wang P., Marsh E.L., Mitchell R.B., Schachtman D.P. (2018). Isolation and Analysis of Microbial Communities in Soil, Rhizosphere, and Roots in Perennial Grass Experiments. J. Vis. Exp..

[14] Edwin N.R., Fitzpatrick A.H., Brennan F., Abram F., O’Sullivan O. (2024). An In-Depth Evaluation of Metagenomic Classifiers for Soil Microbiomes. Environ. Microbiome.

[15] DeFord L., Yoon J.Y. (2024). Soil Microbiome Characterization and Its Future Directions with Biosensing. J. Biol. Eng..

[16] Khalili B., Weihe C., Kimball S., Schmidt K.T., Martiny J.B.H. (2019). Optimization of a Method To Quantify Soil Bacterial Abundance by Flow Cytometry. mSphere.

[17] Liu J., Li J.Q., Feng L., Cao H., Cui Z. (2010). An Improved Method for Extracting Bacteria from Soil for High Molecular Weight DNA Recovery and BAC Library Construction. J. Microbiol..

[18] Lindahl V. (1996). Improved Soil Dispersion Procedures for Total Bacterial Counts, Extraction of Indigenous Bacteria and Cell Survival. J. Microbiol. Methods.

[19] Durán P., Ellis T.J., Thiergart T., Ågren J., Hacquard S. (2022). Climate Drives Rhizosphere Microbiome Variation and Divergent Selection between Geographically Distant Arabidopsis Populations. New Phytol..

[20] Kim D., Song L., Breitwieser F.P., Salzberg S.L. (2016). Centrifuge: Rapid and Sensitive Classification of Metagenomic Sequences. Genome Res..

[21] Quast C., Pruesse E., Yilmaz P., Gerken J., Schweer T., Yarza P., Peplies J., Glöckner F.O. (2013). The SILVA Ribosomal RNA Gene Database Project: Improved Data Processing and Web-Based Tools. Nucleic Acids Res..

[22] Lu Y., Zhou G., Ewald J., Pang Z., Shiri T., Xia J. (2023). MicrobiomeAnalyst 2.0: Comprehensive Statistical, Functional and Integrative Analysis of Microbiome Data. Nucleic Acids Res..

[23] Do T.-X., Huynh V.-P., Le L.-A., Nguyen T.-V., Nguyen-Pham A.-T., Bui-Thi M.-D., Chau-Thi A.-T., Tran S.-N., Nguyen V.-T., Ho-Huynh T.-D. (2021). Microbial Diversity Analysis Using 16S RRNA Gene Amplicon Sequencing of Rhizosphere Soils from Double-Cropping Rice and Rice-Shrimp Farming Systems in Soc Trang, Vietnam. Microbiol. Resour. Announc..

[24] Maretto L., Deb S., Ravi S., Della Lucia M.C., Borella M., Campagna G., Squartini A., Concheri G., Nardi S., Stevanato P. (2023). 16S Metabarcoding, Total Soil DNA Content, and Functional Bacterial Genes Quantification to Characterize Soils under Long-Term Organic and Conventional Farming Systems. Chem. Biol. Technol. Agric..

[25] Varliero G., Lebre P.H., Adams B., Chown S.L., Convey P., Dennis P.G., Fan D., Ferrari B., Frey B., Hogg I.D. (2024). Biogeographic Survey of Soil Bacterial Communities across Antarctica. Microbiome.

[26] Alekseev I., Zverev A., Abakumov E. (2020). Microbial Communities in Permafrost Soils of Larsemann Hills, Eastern Antarctica: Environmental Controls and Effect of Human Impact. Microorganisms.

[27] Ramos L.R., Vollú R.E., Jurelevicius D., Rosado A.S., Seldin L. (2019). Firmicutes in Different Soils of Admiralty Bay, King George Island, Antarctica. Polar Biol..

[28] Enebe M.C., Babalola O.O. (2020). Effects of Inorganic and Organic Treatments on the Microbial Community of Maize Rhizosphere by a Shotgun Metagenomics Approach. Ann. Microbiol..

[29] Schultz J., Argentino I.C.V., Kallies R., Nunes da Rocha U., Rosado A.S. (2022). Polyphasic Analysis Reveals Potential Petroleum Hydrocarbon Degradation and Biosurfactant Production by Rare Biosphere Thermophilic Bacteria From Deception Island, an Active Antarctic Volcano. Front. Microbiol..

[30] Coleine C., Stajich J.E., Pombubpa N., Zucconi L., Onofri S., Canini F., Selbmann L. (2019). Altitude and Fungal Diversity Influence the Structure of Antarctic Cryptoendolithic Bacteria Communities. Environ. Microbiol. Rep..

[31] Davis C.L., Venturelli R.A., Michaud A.B., Hawkings J.R., Achberger A.M., Vick-Majors T.J., Rosenheim B.E., Dore J.E., Steigmeyer A., Skidmore M.L. (2023). Biogeochemical and Historical Drivers of Microbial Community Composition and Structure in Sediments from Mercer Subglacial Lake, West Antarctica. ISME Commun..

[32] Bakermans C., Skidmore M.L., Douglas S., McKay C.P. (2014). Molecular Characterization of Bacteria from Permafrost of the Taylor Valley, Antarctica. FEMS Microbiol. Ecol..

[33] Zheng R., Zhao Y., Wang L., Chang X., Zhang Y., Da X., Peng F. (2016). *Mucilaginibacter antarcticus* Sp. Nov., Isolated from Tundra Soil. Int. J. Syst. Evol. Microbiol..

[34] Karlov D.S., Marie D., Chuvochina M.S., Alekhina I.A., Bulat S.A. (2011). Microbial Communities of Water Column of Lake Radok, East Antarctica, Dominated by Abundant Actinobacterium “Candidatus Planktophila Limnetica”. Microbiology.

[35] da Silva J.P., Veloso T.G.R., Costa M.D., de Souza J.J.L.L., Soares E.M.B., Gomes L.C., Schaefer C.E.G.R. (2024). Microbial Successional Pattern along a Glacier Retreat Gradient from Byers Peninsula, Maritime Antarctica. Environ. Res..

[36] Contreras M.J., Leal K., Bruna P., Nuñez-Montero K., Goméz-Espinoza O., Santos A., Bravo L., Valenzuela B., Solis F., Gahona G. (2023). Commonalities between the Atacama Desert and Antarctica Rhizosphere Microbial Communities. Front. Microbiol..

[37] Zeng Y., Nupur Y., Wu N., Madsen A.M., Chen X., Gardiner A.T., Koblížek M. (2021). *Gemmatimonas groenlandica* Sp. Nov. Is an Aerobic Anoxygenic Phototroph in the Phylum Gemmatimonadetes. Front. Microbiol..

[38] Rawat S.R., Männistö M.K., Bromberg Y., Häggblom M.M. (2012). Comparative Genomic and Physiological Analysis Provides Insights into the Role of Acidobacteria in Organic Carbon Utilization in Arctic Tundra Soils. FEMS Microbiol. Ecol..

[39] Lian T., Ma Q., Shi Q., Cai Z., Zhang Y., Cheng Y., Nian H. (2019). High Aluminum Stress Drives Different Rhizosphere Soil Enzyme Activities and Bacterial Community Structure between Aluminum-Tolerant and Aluminum-Sensitive Soybean Genotypes. Plant Soil.

[40] Oshiki M., Toyama Y., Suenaga T., Terada A., Kasahara Y., Yamaguchi T., Araki N. (2022). N2O Reduction by *Gemmatimonas aurantiaca* and Potential Involvement of Gemmatimonadetes Bacteria in N2O Reduction in Agricultural Soils. Microbes Environ..

[41] Qiao Y., Wang T., Huang Q., Guo H., Zhang H., Xu Q., Shen Q., Ling N. (2024). Core Species Impact Plant Health by Enhancing Soil Microbial Cooperation and Network Complexity during Community Coalescence. Soil Biol. Biochem..

[42] Madhaiyan M., Poonguzhali S., Lee J.S., Senthilkumar M., Lee K.C., Sundaram S. (2010). *Mucilaginibacter gossypii* Sp. Nov. and *Mucilaginibacter gossypiicola* Sp. Nov., Plant-Growth-Promoting Bacteria Isolated from Cotton Rhizosphere Soils. Int. J. Syst. Evol. Microbiol..

[43] Yahya G., Ebada A., Khalaf E.M., Mansour B., Nouh N.A., Mosbah R.A., Saber S., Moustafa M., Negm S., El-Sokkary M.M.A. (2021). Soil-Associated Bacillus Species: A Reservoir of Bioactive Compounds with Potential Therapeutic Activity against Human Pathogens. Microorganisms.

[44] Khan M.S., Gao J., Chen X., Zhang M., Yang F., Du Y., Moe T.S., Munir I., Xue J., Zhang X. (2020). Isolation and Characterization of Plant Growth-Promoting Endophytic Bacteria *Paenibacillus polymyxa* SK1 from *Lilium lancifolium*. Biomed. Res. Int..

[45] Ofek M., Hadar Y., Minz D. (2012). Ecology of Root Colonizing Massilia (Oxalobacteraceae). PLoS ONE.

[46] Da Mota F.F., Gomes E.A., Paiva E., Seldin L. (2005). Assessment of the Diversity of Paenibacillus Species in Environmental Samples by a Novel RpoB-Based PCR-DGGE Method. FEMS Microbiol. Ecol..

[47] Baek J.H., Baek W., Ruan W., Jung H.S., Lee S.C., Jeon C.O. (2022). *Massilia soli* Sp. Nov., Isolated from Soil. Int. J. Syst. Evol. Microbiol..

[48] Foysal M.J., Lisa A.K. (2018). Isolation and Characterization of *Bacillus* Sp. Strain BC01 from Soil Displaying Potent Antagonistic Activity against Plant and Fish Pathogenic Fungi and Bacteria. J. Genet. Eng. Biotechnol..

[49] Lee H., Kim D.U., Park S., Yoon J.H., Ka J.O. (2017). *Massilia chloroacetimidivorans* Sp. Nov., a Chloroacetamide Herbicide-Degrading Bacterium Isolated from Soil. Antonie Van Leeuwenhoek Int. J. Gen. Mol. Microbiol..

[50] Jalilvand N., Akhgar A., Alikhani H.A., Rahmani H.A., Rejali F. (2020). Removal of Heavy Metals Zinc, Lead, and Cadmium by Biomineralization of Urease-Producing Bacteria Isolated from Iranian Mine Calcareous Soils. J. Soil Sci. Plant Nutr..

[51] Zhang X., Gao Y., Zang P., Zhao Y., He Z., Zhu H., Song S., Zhang L. (2019). Study on the Simultaneous Degradation of Five Pesticides by *Paenibacillus polymyxa* from Panax Ginseng and the Characteristics of Their Products. Ecotoxicol. Environ. Saf..

[52] Pal A.K., Sengupta C. (2019). Isolation of Cadmium and Lead Tolerant Plant Growth Promoting Rhizobacteria: *Lysinibacillus varians* and Pseudomonas Putida from Indian Agricultural Soil. Soil Sediment Contam. Int. J..

[53] Brat K., Sedlacek I., Sevcikova A., Merta Z., Laska K., Sevcik P. (2016). Imported Anthropogenic Bacteria May Survive the Antarctic Winter and Introduce New Genes into Local Bacterial Communities. Pol. Polar Res..

[54] de Francisco Martínez P., Morgante V., González-Pastor J.E. (2022). Isolation of Novel Cold-Tolerance Genes from Rhizosphere Microorganisms of Antarctic Plants by Functional Metagenomics. Front. Microbiol..

[55] Coleine C., Albanese D., Ray A.E., Delgado-Baquerizo M., Stajich J.E., Williams T.J., Larsen S., Tringe S., Pennacchio C., Ferrari B.C. (2024). Metagenomics Untangles Potential Adaptations of Antarctic Endolithic Bacteria at the Fringe of Habitability. Sci. Total Environ..

[56] Lebre P.H., Bosch J., Coclet C., Hallas R., Hogg I.D., Johnson J., Moon K.L., Ortiz M., Rotimi A., Stevens M.I. (2023). Expanding Antarctic Biogeography: Microbial Ecology of Antarctic Island Soils. Ecography.

[57] Chong C.W., Silvaraj S., Supramaniam Y., Snape I., Tan I.K.P. (2018). Effect of Temperature on Bacterial Community in Petroleum Hydrocarbon-Contaminated and Uncontaminated Antarctic Soil. Polar Biol..

[58] Chong C.W., Dunn M.J., Convey P., Tan G.Y.A., Wong R.C.S., Tan I.K.P. (2009). Environmental Influences on Bacterial Diversity of Soils on Signy Island, Maritime Antarctic. Polar Biol..

[59] Chong C.W., Pearce D.A., Convey P., Tan G.Y.A., Wong R.C.S., Tan I.K.P. (2010). High Levels of Spatial Heterogeneity in the Biodiversity of Soil Prokaryotes on Signy Island, Antarctica. Soil Biol. Biochem..

[60] Acevedo-Barrios R., Bertel-Sevilla A., Alonso-Molina J., Olivero-Verbel J. (2019). Perchlorate-Reducing Bacteria from Hypersaline Soils of the Colombian Caribbean. Int. J. Microbiol..

[61] Salam L.B., Obayori O.S., Ilori M.O., Amund O.O. (2023). Chromium Contamination Accentuates Changes in the Microbiome and Heavy Metal Resistome of a Tropical Agricultural Soil. World J. Microbiol. Biotechnol..

[62] Ibrahim U.B., Kawo A.H., Yusuf I., Yahaya S. (2021). Physicochemical and Molecular Characterization of Heavy Metal–Tolerant Bacteria Isolated from Soil of Mining Sites in Nigeria. J. Genet. Eng. Biotechnol..

[63] Aburto-Medina A., Adetutu E.M., Aleer S., Weber J., Patil S.S., Sheppard P.J., Ball A.S., Juhasz A.L. (2012). Comparison of Indigenous and Exogenous Microbial Populations during Slurry Phase Biodegradation of Long-Term Hydrocarbon-Contaminated Soil. Biodegradation.

[64] Tong H., Hu M., Li F., Chen M., Lv Y. (2015). Burkholderiales Participating in Pentachlorophenol Biodegradation in Iron-Reducing Paddy Soil as Identified by Stable Isotope Probing. Environ. Sci. Process Impacts.

