# Supplementary material for: Metataxonomics Characterization of Soil Microbiome Extraction Method Using Different Dispersant Solutions
Source: Microorganisms. 2025 Apr 18;13(4):936. doi: 10.3390/microorganisms13040936 (PMC12029719; doi:10.3390/microorganisms13040936)
Supplement: Supplementary file 1 [file microorganisms-13-00936-s001.zip › microorganisms-3545148-supplementary.pdf]

microorganisms

Communication

Metataxonomics Characterization of Soil Microbiome Extraction Method Using Different Dispersant Solutions

David Madariaga-Troncoso [1], Isaac Vargas [2], Dorian Rojas-Villalta [3], Michel Abanto [4,*] and Kattia Núñez-Montero [1,*]

[1] Facultad de Ciencias de La Salud, Instituto de Ciencias Aplicadas, Universidad Autónoma de Chile, Temuco 4810101, Chile; david.madariaga@cloud.uautonoma.cl
[2] Escuela de Biología, Instituto Tecnológico de Costa Rica, Cartago 30101, Costa Rica; isaacvv131200@gmail.com
[3] Cellular and Molecular Biology Research Center, Universidad de Costa Rica, San José 11501, Costa Rica; rojasvillaltadorian@gmail.com
[4] Núcleo Científico y Tecnológico en Biorecursos (BIOREN), Universidad de La Frontera, Avenida Francisco Salazar, Temuco 4811230, Chile
* Correspondence: michel.abanto@ufrontera.cl (M.A.); kattia.nunez@uautonoma.cl (K.N.-M.)

Academic Editor: Nikolay Vassilev

Received: 7 March 2025
Revised: 7 April 2025
Accepted: 15 April 2025
Published: 18 April 2025

Citation: Madariaga-Troncoso, D.; Vargas, I.; Rojas-Villalta, D.; Abanto, M.; Núñez-Montero, K. Metataxonomics Characterization of Soil Microbiome Extraction Method Using Different Dispersant Solutions. *Microorganisms* **2025**, *13*, 936. https://doi.org/10.3390/microorganisms13040936

Copyright: © 2025 by the authors. Licensee MDPI, Basel, Switzerland. This article is an open access article distributed under the terms and conditions of the Creative Commons Attribution (CC BY) license (https://creativecommons.org/licenses/by/4.0/).

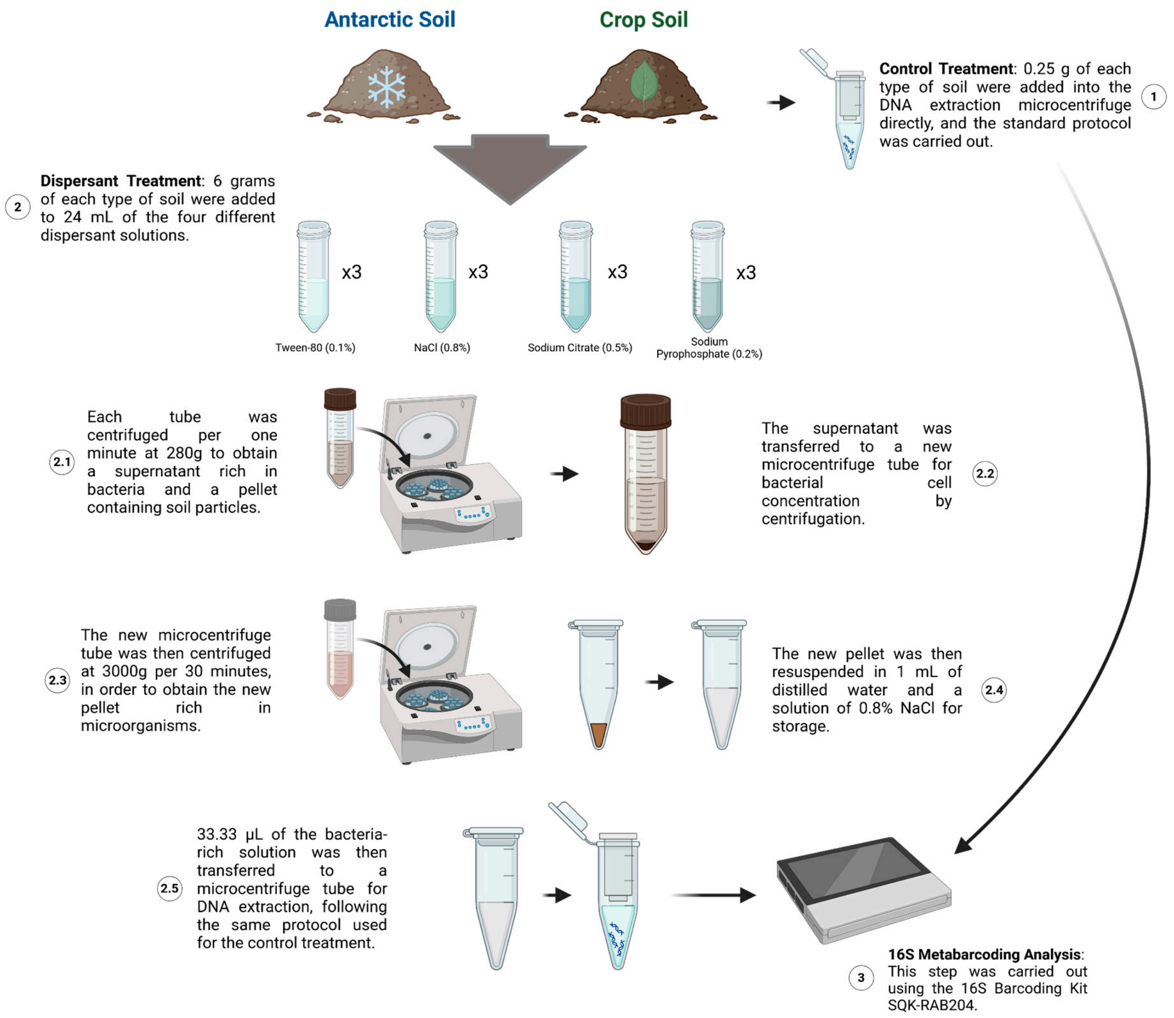

Figure S1. Antarctic and Agricultural soil microbiome DNA extraction protocol using different dispersant solutions. The workflow shows both control (1) and dispersant treatments (2) with four different dispersant solutions in triplicated assays, followed by sequential centrifugation steps (2.1-2.3), bacterial pellet storage (2.4), and final DNA extraction (2.5). The extracted DNA was subsequently processed for 16S rRNA metataxonomic analysis (3) using the 16S Barcoding SQK-RAB204 Kit to characterize the bacterial communities. Created using BioRender.com.

Table S1. Chemical parameters of the soil samples included in this study

	**Antarctic Soil**	**Crop Soil**
P (mg/kg)	21	6
K (mg/kg)	239	407
pH (en agua)	7.17	5.89
Materia orgánica (%)	2	13
K (cmol+/kg)	0.61	1.04
Na (cmol+/kg)	1.8	0.1
Ca (cmol+/kg)	17.44	11.17
Mg (cmol+/kg)	15.55	2.46
Al (cmol+/kg)	0.06	0.16
Saturación de Al (%)	0.17	1.07
CICE (cmol+/kg)	35.46	14.93
S. Bases (cmol+/kg)	35.4	14.77
B (mg/kg)	0.25	0.48
Zn (mg/kg)	0.23	0.74
Cu (mg/kg)	2.12	0.87
Fe (mg/kg)	30.3	57
Mn (mg/kg)	0.01	5.9
S (mg/kg)	3	7
Al Ext. (mg/kg)	122	985
pH CaCl2	5.99	4.91

Table S2. Alpha diversity values for crop and Antarctic soil samples.

Soil	Index	Tween80	Sodium chloride	Sodium citrate	Pyro-phosphate	Control
**Crop**	Chao1	434.77 ± 30.27	382.71 ± 18.77	380.31 ± 18.85	420.20 ± 24.15	308.76 ± 16.08
	Shannon	3.27 ± 0.39	4.24 ± 0.17	3.29 ± 0.28	3.59 ± 0.37	3.24 ± 0.26
**Antarctic**	Chao1	553.59	69.199	276.8	276.8	3.07
	Shannon	3.99 ± 0.13	3.86 ± 0.16	4.04 ± 0.07	4.07 ± 0.10	3.58 ± 0.82

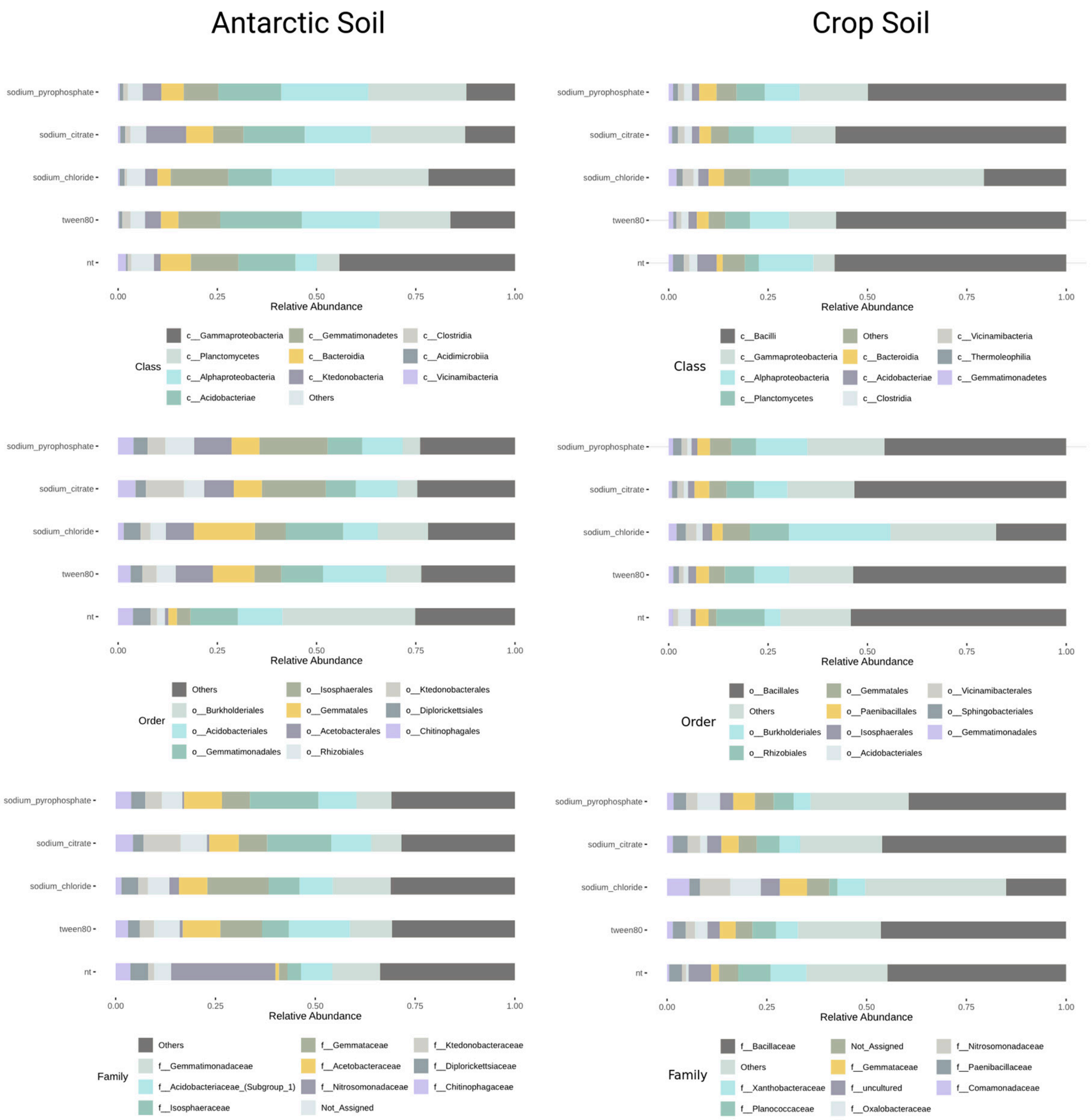

Figure S2. **Taxonomic composition of bacterial communities at Class, Order, and Family levels in Antarctic and crop soils under different dispersant treatments.** Relative abundance of bacterial taxa at Class, Order, and Family taxonomic ranks in Antarctic soil (left panels) and crop soil (right panels) after extraction using different dispersant solutions: sodium pyrophosphate, sodium citrate, sodium chloride, Tween80, and no-treatment control (nt). The data illustrates the distinct taxonomic compositions between soil types and the effects of different dispersant treatments on the extracted microbial communities. This analysis complements the phylum and genus-level data shown in Figure 1.

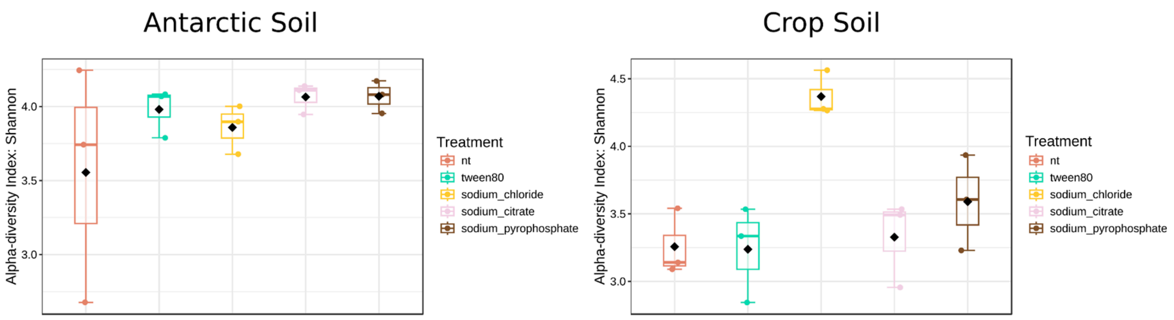

Figure S3. Alpha diversity analysis using Shannon index for Antarctic and crop soils treated with different dispersant solutions. Shannon diversity index values for bacterial communities in Antarctic soil (left) and crop soil (right) after treatment with different dispersant solutions (Tween-80, sodium chloride, sodium citrate, and sodium pyrophosphate) compared to no-treatment control (nt).

Table S3. Linear Discriminant Analysis Effect Size (LEfSe) of Antarctic soil samples.

Taxon	P-value	FDR	Control	Tween80	Sodium chloride	Sodium citrate	Pyro-phos-phate	LDA Score
**p__Abditibacteriota**	0.043115	0.43164	1314.8	1937.6	3114	830.39	276.8	3.15
**p__Bacteroidota**	0.049067	0.43164	77157	43457	36399	68092	55359	4.31
**p__Nitrospirota**	0.049804	0.43164	2422	553.59	69.199	276.8	276.8	3.07
**c__Kapabacteria**	0.017115	0.33645	484.4	899.59	2283.6	484.4	69.199	3.04
**c__Gammaproteobacteria**	0.018635	0.33645	440800	161100	219500	124140	117020	5.21
**c__Phycisphaerae**	0.020298	0.33645	761.19	2076	899.59	4567.2	5466.7	3.37
**c__Clostridia**	0.022347	0.33645	8234.7	20206	5951.1	12041	12179	3.85
**c__Nitrospiria**	0.029808	0.33645	2422	484.4	0	138.4	207.6	3.08
**c__Bacilli**	0.033728	0.33645	12248	5466.7	11418	3806	2422	3.69
**c__Abditibacteria**	0.043115	0.33645	1314.8	1937.6	3114	830.39	276.8	3.15
**c__Bacteroidia**	0.047733	0.33645	76673	42558	34115	67608	55290	4.33
**c__Oligoflexia**	0.049883	0.33645	1314.8	1799.2	2006.8	3390.8	3252.4	3.02
**o__Kapabacteriales**	0.017115	0.35475	484.4	899.59	2283.6	484.4	69.199	3.04
**o__Tepidisphaerales**	0.017832	0.35475	761.19	2006.8	830.39	4152	5397.6	3.37
**o__Clostridiales**	0.017847	0.35475	2906.4	19722	5259.2	10726	11764	3.92
**o__Burkholderiales**	0.018635	0.35475	334160	87122	127670	50585	40136	5.17
**o__Xanthomonadales**	0.018635	0.35475	51484	23389	28372	6850.7	5674.3	4.36
**o__EV818SWSAP88**	0.019751	0.35475	0	415.2	207.6	2006.8	2283.6	3.06
**o__Pirellulales**	0.019758	0.35475	346	3183.2	2006.8	1591.6	1522.4	3.15
**o__Cytophagales**	0.020263	0.35475	1176.4	899.59	346	2768	2352.8	3.08
**o__Salinisphaerales**	0.022876	0.35475	484.4	1107.2	1038	2698.8	5190	3.37
**o__Bacillales**	0.024063	0.35475	4290.4	2214.4	6643.1	1522.4	553.59	3.48
**o__Nitrospirales**	0.029808	0.35475	2422	484.4	0	138.4	207.6	3.08
**o__Solibacterales**	0.030226	0.35475	6989.1	2906.4	2214.4	5951.1	6850.7	3.38
**o__Gemmatales**	0.034684	0.35475	21037	106220	153480	69061	71414	4.82
**o__Paenibacillales**	0.038039	0.35475	691.99	2975.6	4290.4	2214.4	1730	3.26
**o__Rhizobiales**	0.039988	0.35475	20137	47748	38682	52246	72452	4.42
**o__Holosporales**	0.040387	0.35475	1384	5951.1	13425	3875.2	3460	3.78
**o__Isosphaerales**	0.040993	0.35475	34530	68438	77503	162340	174800	4.85
**o__Abditibacteriales**	0.043115	0.35475	1314.8	1937.6	3114	830.39	276.8	3.15
**o__Paracaedibacterales**	0.047263	0.35475	0	1176.4	1107.2	138.4	346	2.77
**o__Caulobacterales**	0.048395	0.35475	9341.9	21798	11072	20760	27957	3.97
**o__GOUTB8**	0.049589	0.35475	0	138.4	138.4	207.6	346	2.24

Table S4. Linear Discriminant Analysis Effect Size (LEfSe) of Crop soil samples.

Taxon	P-value	FDR	Control	Tween80	Sodium chloride	Sodium citrate	Pyro-phos-phate	LDA Score
**p__Bdellovibrionota**	0.031216	0.2674	2798.7	2726.9	6960.9	4377.5	5382.1	3.33
**c__Kapabacteria**	0.015548	0.33514	0	287.05	574.09	430.57	143.52	2.46
**c__Gammaproteobacteria**	0.021296	0.33514	52530	114170	346750	111230	167420	5.17
**c__Alphaproteobacteria**	0.028502	0.33514	136420	97811	140440	93506	86903	4.43
**c__Omnitrophia**	0.037648	0.33514	0	574.09	861.14	143.52	430.57	2.64
**o__Pseudomonadales**	0.012818	0.3486	3229.3	5166.8	28059	7176.2	17797	4.09
**o__Kapabacteriales**	0.015548	0.3486	0	287.05	574.09	430.57	143.52	2.46
**o__Burkholderiales**	0.023063	0.3486	39900	86042	254320	83459	123930	5.03
**o__Rhodospirillales**	0.026183	0.3486	430.57	1435.2	5095.1	1865.8	1937.6	3.37
**o__Caulobacterales**	0.03007	0.3486	430.57	2081.1	4449.2	2511.7	2009.3	3.3
**o__Rhizobiales**	0.030151	0.3486	120630	73197	98385	69035	61141	4.47
**o__Flavobacteriales**	0.03027	0.3486	430.57	932.9	1435.2	2583.4	3875.1	3.24
**o__Xanthomonadales**	0.034996	0.3486	3444.6	12558	15213	9759.6	9185.5	3.77
**o__Reyranellales**	0.036988	0.3486	4305.7	7319.7	11267	8180.8	7319.7	3.54
**o__Omnitrophales**	0.037648	0.3486	0	574.09	861.14	143.52	430.57	2.64
**o__uncultured**	0.045708	0.3486	5166.8	3301	6889.1	3516.3	3229.3	3.26
**o__Diplorickettsiales**	0.045746	0.3486	2152.9	5525.7	33728	5095.1	7176.2	4.2
**o__Frankiales**	0.046257	0.3486	932.9	0	0	358.81	143.52	2.67
**o__Legionellales**	0.046526	0.3486	861.14	861.14	4879.8	2511.7	2439.9	3.3
**o__Sphingobacteriales**	0.046895	0.3486	1004.7	13922	24758	13132	21529	4.07
**f__Sporolactobacillaceae**	0.0084695	0.28341	0	0	0	287.05	574.09	2.46
**f__NS11_12_marine_group**	0.012	0.28341	215.29	4162.2	14281	4377.5	8396.1	3.85
**f__Pseudomonadaceae**	0.015698	0.28341	502.33	2726.9	17438	2511.7	11051	3.93
**f__Pseudohongiellaceae**	0.015751	0.28341	0	358.81	1937.6	502.33	1219.9	2.99
**f__Xanthomonadaceae**	0.016623	0.28341	2081.1	6315	9759.6	5166.8	3946.9	3.58
**f__Rhodospirillaceae**	0.017351	0.28341	143.52	789.38	3372.8	861.14	1148.2	3.21
**f__Planococcaceae**	0.021484	0.28341	83531	63079	20596	58127	50879	4.5
**f__Oxalobacteraceae**	0.022738	0.28341	4951.6	31503	74345	17940	54180	4.54
**f__Schlesneriaceae**	0.025046	0.28341	287.05	143.52	1363.5	789.38	287.05	2.79
**f__Comamonadaceae**	0.02525	0.28341	5525.7	13419	55400	14424	15716	4.4
**f__Rhodocyclaceae**	0.025271	0.28341	645.86	1363.5	5310.4	1435.2	3014	3.37
**f__Flavobacteriaceae**	0.03027	0.28341	430.57	932.9	1435.2	2583.4	3875.1	3.24
**f__Reyranellaceae**	0.036988	0.28341	4305.7	7319.7	11267	8180.8	7319.7	3.54
**f__Polyangiaceae**	0.037243	0.28341	2224.6	717.62	430.57	861.14	1722.3	2.95
**f__Omnitrophaceae**	0.037648	0.28341	0	574.09	861.14	143.52	430.57	2.64
**f__Xanthobacteraceae**	0.038149	0.28341	88841	53319	69681	51166	42268	4.37
**f__Elsteraceae**	0.040578	0.28341	143.52	287.05	789.38	71.762	502.33	2.56
**f__Caulobacteraceae**	0.040935	0.28341	287.05	1937.6	4233.9	2296.4	1937.6	3.3
**f__Hungateiclostridiaceae**	0.043424	0.28341	1291.7	358.81	430.57	502.33	1291.7	2.67
**f__Diplorickettsiaceae**	0.045746	0.28341	2152.9	5525.7	33728	5095.1	7176.2	4.2
**f__Acidothermaceae**	0.046257	0.28341	932.9	0	0	358.81	143.52	2.67
**f__Legionellaceae**	0.046526	0.28341	861.14	861.14	4879.8	2511.7	2439.9	3.3

**f__Nitrosomonadaceae**	0.047078	0.28341	11123	23897	75637	31216	27987	4.51
**f__Family_XI**	0.048813	0.28341	358.81	0	0	215.29	287.05	2.26
**f__Methylophilaceae**	0.049129	0.28341	0	717.62	5095.1	574.09	1578.8	3.41
**g__Lautropia**	0.0078026	0.33389	0	0	287.05	0	0	2.16
**g__Sporolactobacillus**	0.0084695	0.33389	0	0	0	287.05	574.09	2.46
**g__Curvibacter**	0.01542	0.33389	0	71.762	502.33	0	287.05	2.4
**g__Pseudomonas**	0.015698	0.33389	502.33	2726.9	17438	2511.7	11051	3.93
**g__BIyi10**	0.015751	0.33389	0	358.81	1937.6	502.33	1219.9	2.99
**g__Limnohabitans**	0.017287	0.33389	0	143.52	3014	789.38	645.86	3.18
**g__Oceanobacillus**	0.01813	0.33389	1937.6	1148.2	215.29	2152.9	1148.2	2.99
**g__Clostridium_sensu_ stricto_1**	0.018329	0.33389	3659.8	1794	574.09	2152.9	3516.3	3.19
**g__Arenimonas**	0.019691	0.33389	1578.8	3229.3	6243.3	2726.9	1794	3.37
**g__Psychrobacillus**	0.020046	0.33389	10764	6530.3	2439.9	5310.4	4449.2	3.62
**g__Herbaspirillum**	0.020409	0.33389	215.29	645.86	3014	287.05	1937.6	3.15
**g__GOUTA6**	0.020772	0.33389	358.81	2081.1	7032.7	5597.4	5166.8	3.52
**g__Rhodoferax**	0.022446	0.33389	0	1076.4	9113.7	1507	1507	3.66
**g__Massilia**	0.023391	0.33389	3157.5	17366	39971	10047	26193	4.27
**g__Lysinibacillus**	0.023859	0.33389	20165	10908	3372.8	9185.5	9257.3	3.92
**g__Schlesneria**	0.025046	0.33389	287.05	143.52	1363.5	789.38	287.05	2.79
**Not_Assigned**	0.025463	0.33389	153930	115540	191100	132830	142300	4.58
**g__MND1**	0.027324	0.33389	3229.3	13204	31288	15501	14998	4.15
**g__Caryophanon**	0.028956	0.33389	1865.8	1937.6	215.29	1507	717.62	2.94
**g__Bacteriovorax**	0.028962	0.33389	0	143.52	574.09	0	430.57	2.46
**g__Janthinobacterium**	0.029783	0.33389	0	1865.8	4090.4	1148.2	3875.1	3.31
**g__Flavobacterium**	0.03027	0.33389	430.57	932.9	1435.2	2583.4	3875.1	3.24
**g__Duganella**	0.03172	0.33389	143.52	3516.3	7176.2	717.62	8396.1	3.62
**g__Ralstonia**	0.032373	0.33389	0	0	574.09	0	143.52	2.46
**g__Bradyrhizobium**	0.033818	0.33389	18873	4664.5	9257.3	5669.2	4592.8	3.85
**g__Methylotenera**	0.036814	0.33389	0	430.57	3085.8	215.29	1219.9	3.19
**g__Halolactibacillus**	0.037524	0.33389	215.29	645.86	71.762	358.81	645.86	2.46
**g__Candidatus_Omnitrophus**	0.037648	0.33389	0	574.09	861.14	143.52	430.57	2.64
**g__Devosia**	0.038144	0.33389	215.29	1794	2296.4	717.62	2224.6	3.02
**g__Pajaroellobacter**	0.038688	0.33389	1650.5	430.57	143.52	645.86	1363.5	2.88
**g__Polaromonas**	0.038851	0.33389	143.52	574.09	5740.9	645.86	1219.9	3.45
**g__Caenimonas**	0.040961	0.33389	71.762	1076.4	2152.9	717.62	502.33	3.02
**g__Caulobacter**	0.042183	0.33389	0	502.33	717.62	430.57	143.52	2.56
**g__Reyranella**	0.04257	0.33389	4305.7	7247.9	11051	8037.3	7176.2	3.53
**g__Acidovorax**	0.044658	0.33389	71.762	645.86	2511.7	789.38	717.62	3.09
**g__Gemmatimonas**	0.046118	0.33389	4808	2798.7	6386.8	2798.7	4736.3	3.25
**g__Acidothermus**	0.046257	0.33389	932.9	0	0	358.81	143.52	2.67
**g__Legionella**	0.046526	0.33389	861.14	861.14	4879.8	2511.7	2439.9	3.3
**g__Asticcacaulis**	0.047046	0.33389	0	143.52	358.81	287.05	358.81	2.26
**g__Planomicrobium**	0.04749	0.33389	1507	1076.4	71.762	861.14	645.86	2.86
**g__Pelomonas**	0.048037	0.33389	0	645.86	1363.5	143.52	215.29	2.83

**g__Tissierella**	0.048813	0.33389	358.81	0	0	215.29	287.05	2.26

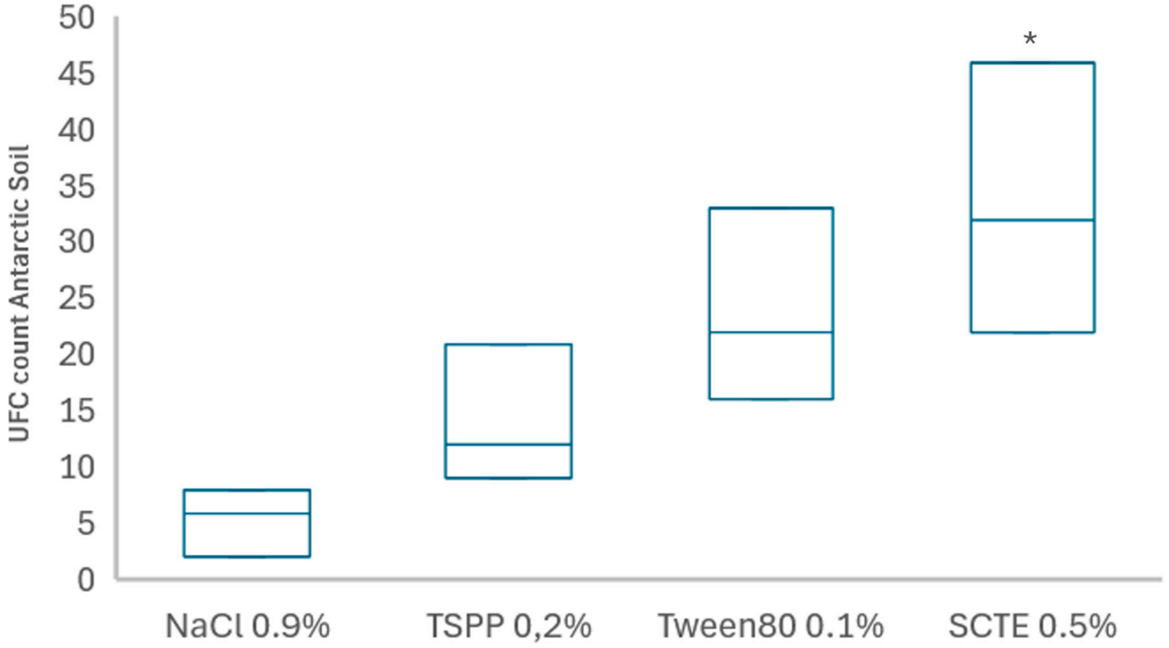

Figure S4. Colony-forming unit (CFU) counts in Antarctic soil samples. UFC = colony-forming units (Spanish acronym), NaCl = sodium chloride treatment, TSPP = Pyrophosphate treatment, SCTE = sodium citrate treatment. Those treatments with a significant difference ($p<0.05$) are indicated with an asterisk (*).

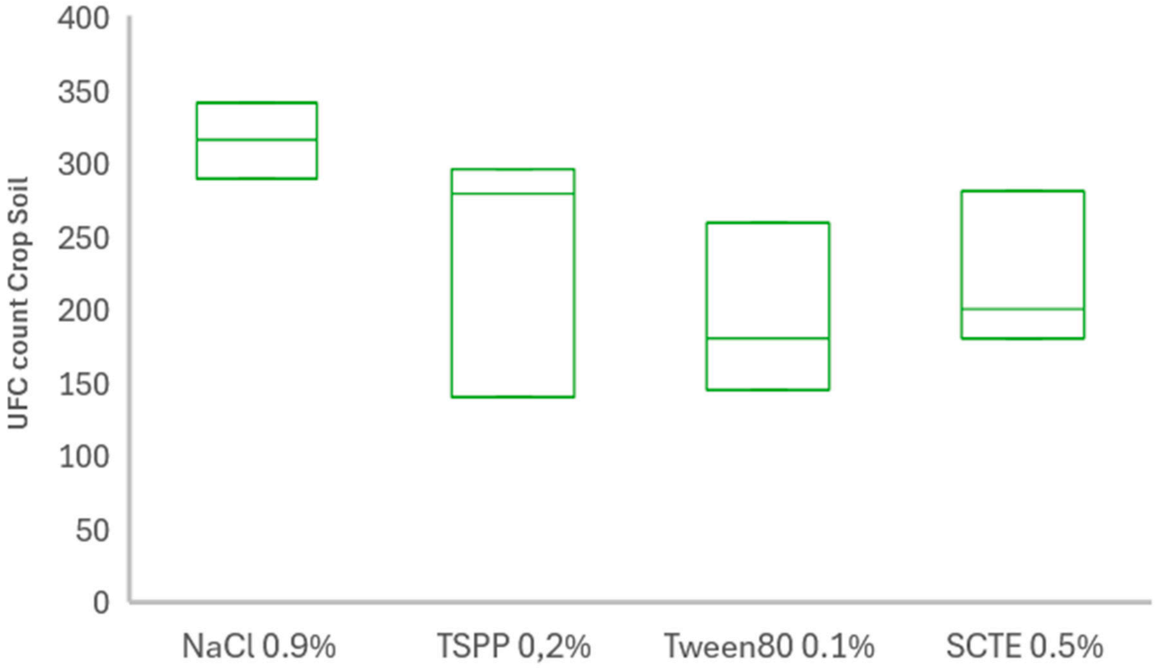

Figure S5. Colony-forming unit (CFU) counts in crop soil samples. UFC = colony-forming units (Spanish acronym), NaCl = sodium chloride treatment, TSPP = Pyrophosphate treatment, SCTE = sodium citrate treatment. No significant differences were present among the treatment ($p>0.05$)

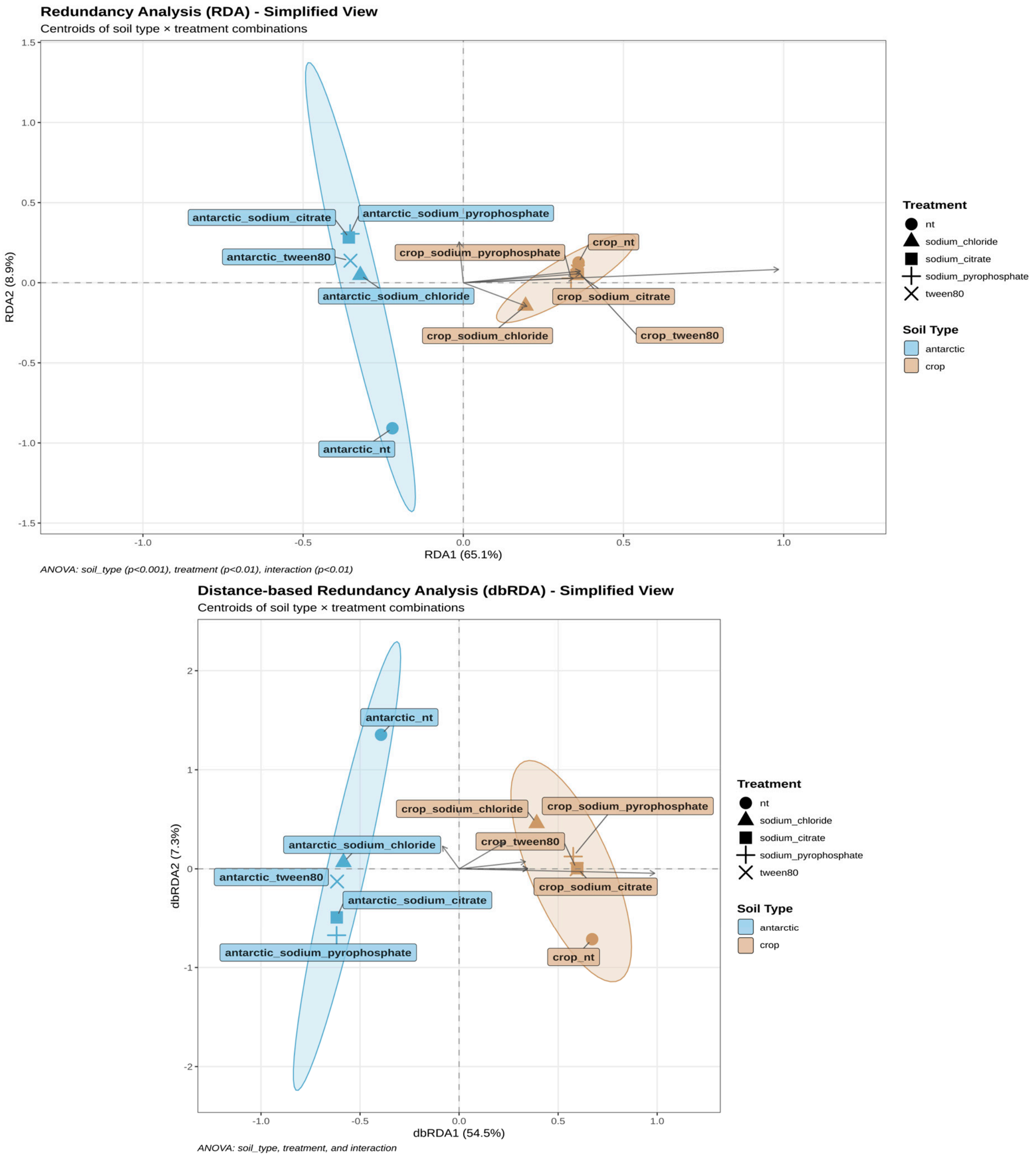

Figure S6. Redundancy Analysis (RDA) and Distance-based Redundancy Analysis (dbRDA) of microbial communities in antarctic and crop soils under different extraction treatments. Ordination plots showing the relationship between soil types (antarctic = blue, crop = tan) and extraction treatments (nt = circles, sodium_chloride = triangles, sodium_citrate = squares, sodium_pyrophosphate = plus signs, tween80 = X signs). (A) RDA of Hellinger-transformed abundance data explains 65.1% variation on axis 1. Statistical significance by ANOVA: soil type ($F = 87.25$, $p = 0.001$), treatment ($F = 3.89$, $p = 0.007$), and interaction ($F = 3.84$, $p = 0.007$). (B) dbRDA explains 54.5% variation on axis 1. Statistical significance by ANOVA: soil type ($F = 47.49$, $p = 0.001$), treatment ($F = 2.79$, $p = 0.006$), and interaction ($F = 2.63$, $p = 0.007$). Ellipses represent 95% confidence intervals for each soil type, and points represent treatment centroids.

Disclaimer/Publisher's Note: The statements, opinions and data contained in all publications are solely those of the individual author(s) and contributor(s) and not of MDPI and/or the editor(s). MDPI and/or the editor(s) disclaim responsibility for any injury to people or property resulting from any ideas, methods, instructions or products referred to in the content.
